# Axillary lymphadenopathy as the initial manifestation in ANCA-associated systemic vasculitis: A case report

**DOI:** 10.1097/MD.0000000000034218

**Published:** 2023-07-21

**Authors:** Jie Du, Hongyue Wang, Lili Zhang, Hongyu Li, Shuang Li, Chao Zhang, Fangfang Sun, Lirong Zhao

**Affiliations:** a Department of Electrical Diagnosis, the First Bethune Hospital of Jilin University, Changchun, China; b Department of Nephrology, the First Bethune Hospital of Jilin University, Changchun, China.

**Keywords:** anti-neutrophil cytoplasmic antibody, axillary lymphadenopathy, case report, kidney damage

## Abstract

The anti-neutrophil cytoplasmic antibody (ANCA) associated vasculitides are a collection of relatively rare autoimmune diseases characterized by the presence of ANCAs, predominantly against myeloperoxidase and proteinase 3. Multiple organs and systems are involved, but superficial lymph node involvement is rarely reported.

**Case presentation:**

A 31-year-old woman initially presented with bilateral axillary lymphadenopathy and that the hilums were not clear. We report the rare case of a patient who presented with an ANCA-associated systemic vasculitis whose initial manifestation was axillary lymphadenopathy. The axillary lymph node needle biopsy specimens had reactive hyperplasia. One year later, the bilateral inguinal lymph nodes had similar morphological and structural changes, and laboratory test results showed renal insufficiency. A renal biopsy revealed the presence of sclerotic glomeruli, crescentic glomeruli, and fibrous crescentic glomeruli, but no deposition of immunocomplex or complement. Finally, the patient was treated with prednisone and mycophenolate mofetil. As the laboratory indicators normalized, so did the sizes of the axillary lymph nodes. A subsequent laboratory examination showed that in addition to urine protein all indicators had normalized, ultrasonography showed slight enlargement of unilateral axillary lymph nodes and normal hilum structure.

**Conclusions:**

Superficial lymphadenopathy is very rare in ANCA-associated systemic vasculitis. Studying this case improves our understanding of the initial manifestations of ANCA-associated vasculitis and may help provide accurate early diagnosis, thus allowing timely treatment and improved patient prognosis.

## 1. Introduction

The anti-neutrophil cytoplasmic antibody (ANCA) associated vasculitides (AAV) are a collection of relatively rare autoimmune diseases that are characterized by systemic necrotizing vasculitis of small vessels, particularly small arteries, arterioles, capillaries, and venules, and ANCAs, predominantly anti-myeloperoxidase and anti-proteinase 3, but little or no immune complex formation.^[1]^ The pathological features of AAV are full-layer inflammation, necrosis, and granuloma formation in small vessels. AAV affects multiple organs and systems, especially the upper airways, lungs, and kidneys. Lymphadenopathy is a rare presentation in these patients. Although previous studies reported mediastinal and hilar lymphadenopathy in patients with AAV,^[2–5]^ there are only rare reports of superficial lymphadenopathy. Here, we report a patient with AAV whose initial manifestation was axillary lymphadenopathy.

## 2. Case report

**Figure 1.**
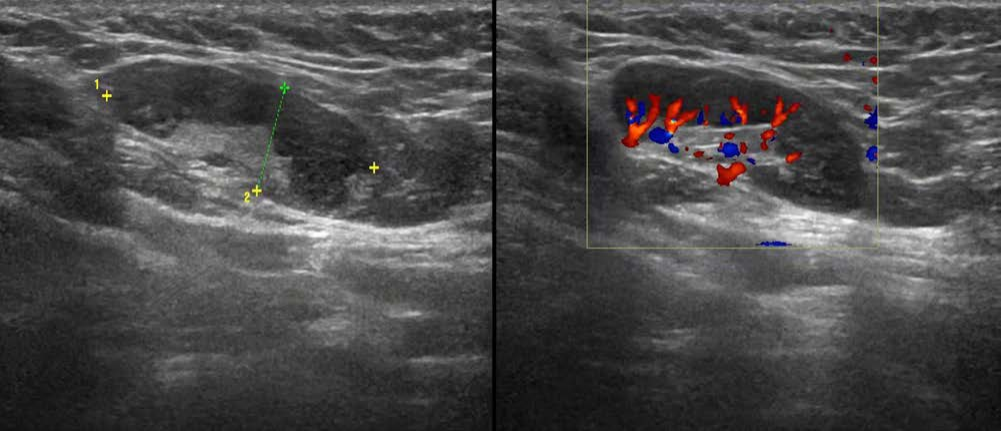
Transverse gray-scale ultrasonography (left), showing that the cortical echo of the right axillary lymph node was decreased and had uneven thickening. Color Doppler ultrasonography (right) showing hilar vascularity.

**Figure 2.**
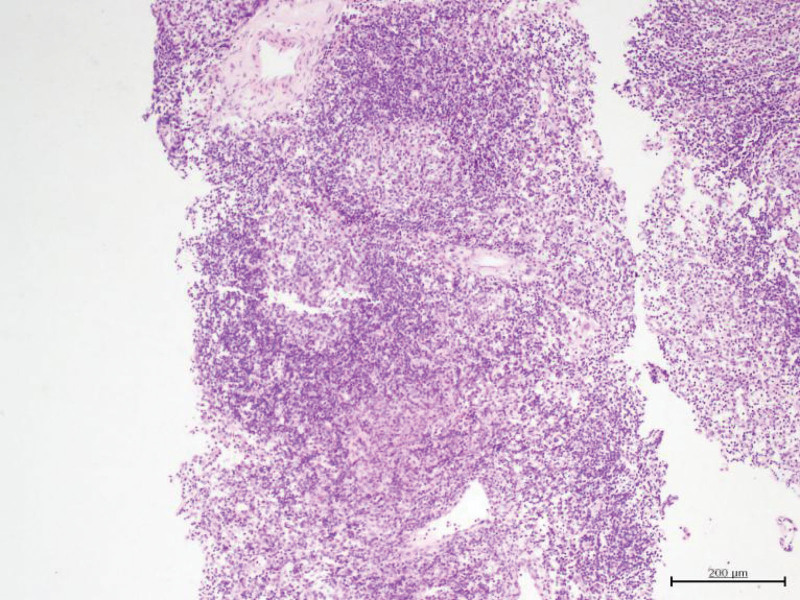
Histology of the axillary lymph node biopsy specimen, showing reactive hyperplasia (H&E staining; original magnification 200×). H&E = hematoxylin-eosin.

A 31-year-old woman initially presented with bilateral axillary lymphadenopathy. A physical examination indicated these lymph nodes had maximal diameters more than 2 cm. Ultrasonography showed that the cortical echoes of some lymph nodes were decreased with uneven thickening and that the hilums were not clear (Fig. 1). Color Doppler ultrasonography showed hilar vascularity. She had no abnormal nodules in the breasts, and no abnormalities in other examinations. At that time, the doctor recommended a needle biopsy of the axillary lymph nodes to clarify their nature and origin. The pathological results were consistent with reactive hyperplasia (Fig. 2). Because of these results and because the patient reported no discomfort, the doctor suggested follow-up by regular color Doppler ultrasonography.

**Figure 3.**
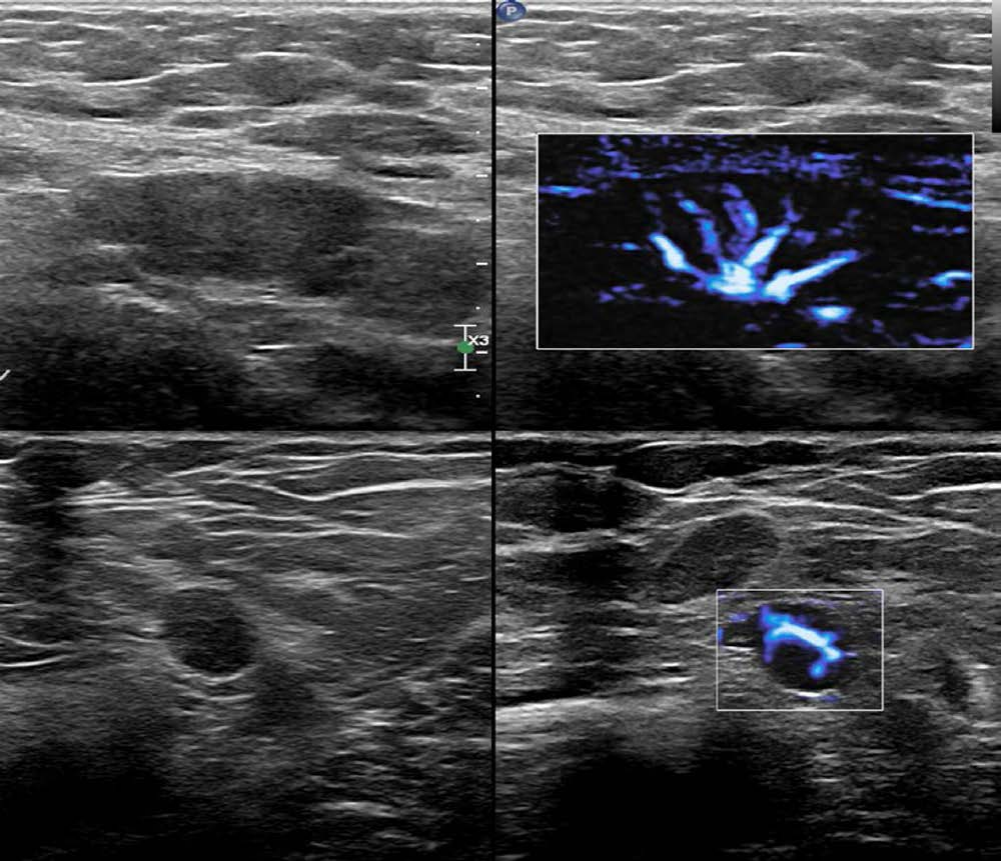
Transverse gray-scale ultrasonography (top left), showing axillary lymph nodes that were round and without hilums. Color Doppler ultrasonography (right), showing abundant hilar vascularity (top right). Bilateral inguinal lymph nodes had similar morphological and structural changes (bottom left and right).

One year later, a physical examination indicated bilateral axillary lymph nodes that had more obvious swelling and were more abundant, with the maximum diameter approaching 3 cm. Their shape and structure had also changed, in that some of them were round and some of the hilums were not clear. Color Doppler ultrasonography showed abundant hilar vascularity. The bilateral inguinal lymph nodes had similar morphological and structural changes (Fig. 3).

Urinalysis showed qualitative urine protein (+++) and occult blood (+++). A physical examination indicated hematuria, but no evidence of edema, headache, dizziness, frequent urination, urgent urination, or urination pain. Laboratory measurements indicated the C-reactive protein was 65 mg/L (high), red blood cell count was 3.13 × 10^12^/L (low), hemoglobin was 93 g/L (low), total leukocyte count was 19.29 × 10^9^/L (0.1% eosinophils;), albumin was 28.7 g/L (low), creatinine was 153 µmol/L (high), serum urea nitrogen was 9.9 µmol/L (high), uric acid 421 was µmol/L (high), 24-h urine protein was 2867.44 mg (high), immunoglobulin G was 21.40 g/L (low), complement C4 was 0.068 g/L (low), anti-SSA was 86.28 U/mL (high), and anti-nucleosome antibodies was 24.75 U/mL (high). The anti-myeloperoxidase ANCA test was positive (perinuclear pattern) with a titer of 1:100, but the anti-proteinase 3 ANCA test was negative. The levels of rheumatoid factor, anti-cyclic citrullinated peptide antibody, and antistreptohaemolysin O were all normal.

Electrocardiography indicated sinus tachycardia, but no abnormalities were evident on a chest X-ray. Chest computed tomography showed slight inflammation in the lingual lobe and lower lobe of the left lung. During the course of disease, the patient developed a cough and expectoration, but no fever. Based on these examination results, we made a preliminary diagnosis of AAV. Because the patient had evidence of renal dysfunction, we also considered kidney involvement and therefore performed a renal biopsy.

**Figure 4.**
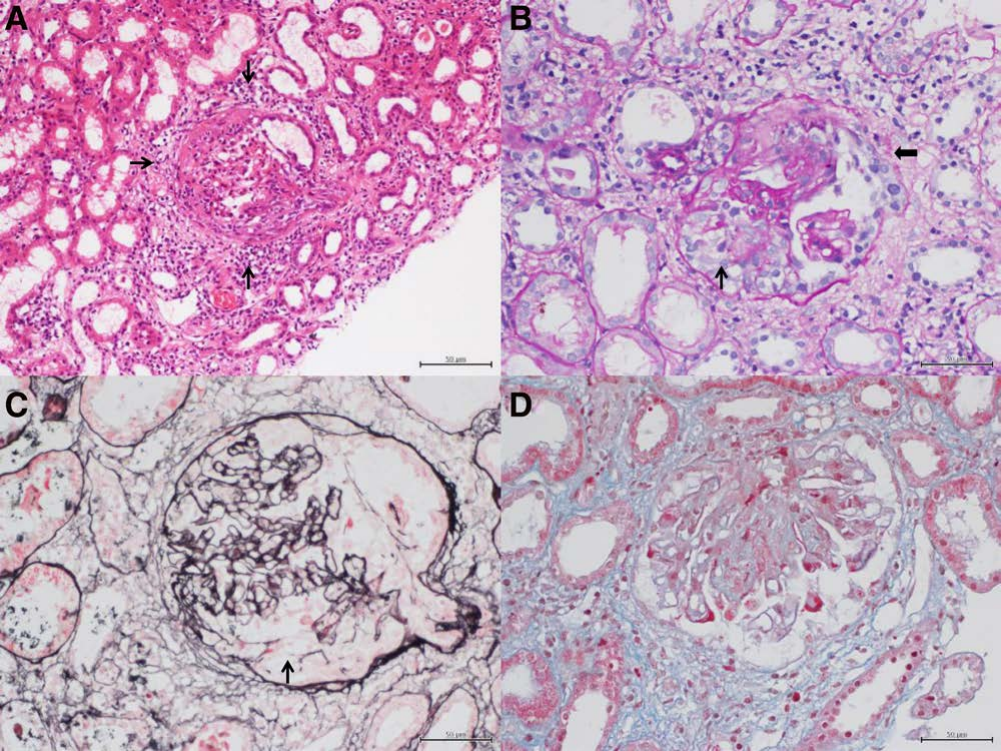
Histopathological examination of a kidney biopsy sample. (A) Diffuse infiltration of inflammatory cells (arrows) around the glomerulus (H&E staining, original magnification: 200×). (B and C) The basement membrane of Baumann’s capsule was discontinuous (thicked black arrow) and there were cellular crescents (black arrow, PAS staining, original magnification: 400×). (D) There was no evidence of polyglobulin deposition (PASM-Masson staining, original magnification: 400×). H&E = hematoxylin-eosin, PASM = periodic acid-silver metheramine.

The renal biopsy yielded 35 glomeruli, 2 with sclerosis, 7 with cellular crescents, and 6 with fibrous crescents (Fig. 4). Immunofluorescence staining showed focal distribution of immunoglobulin M, complement 3, and complement C1q in the mesangial region. The results were negative for immunoglobulin G, immunoglobulin A, complement 4, and fibrinoid. There was no deposition of immunocomplex or complement in the basal membranes of the renal tubules. There was also no immunocomplex or complement deposition in the hilar, vascular, or intertubule capillaries or the interstitial vessels. A test for urinary cylinders was negative. These biopsy results supported the diagnosis of AAV with renal damage.

**Figure 5.**
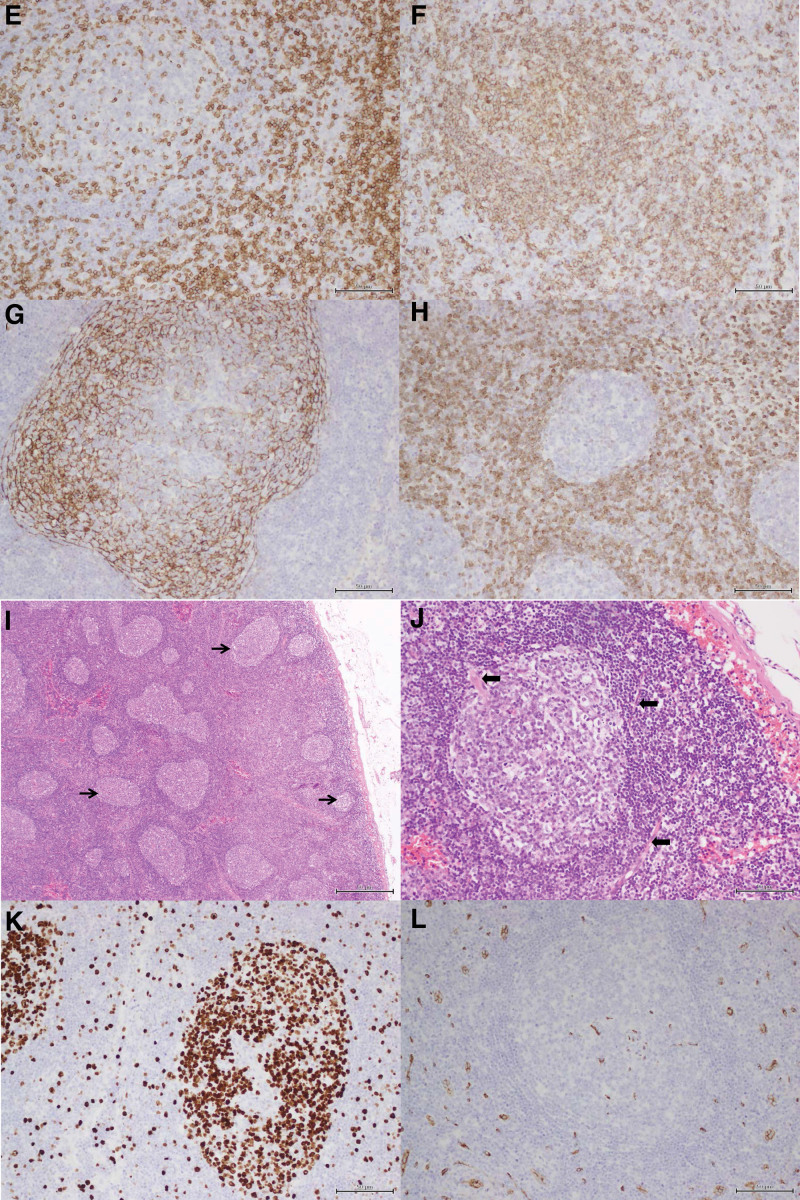
Histopathological examination of axillary lymph nodes. (A and B) Follicular hyperplasia to various extent (black arrows), enlarged germinal centers, and vascular hyperplasia (thicked black arrows; H&E staining; original magnification: 100× in A and 400× in B). (C–H) Immunohistochemical staining for ki67, CD34, CD3, CD20, CD21, BCL-2 (original magnification: 200×). H&E = hematoxylin-eosin.

To further clarify the nature of the axillary lymph nodes, 2 lymph nodes were removed surgically. Morphological and immunohistochemical analysis showed reactive follicular hyperplasia and vascular proliferation (Fig. 5). We administered prednisone (65 mg/d) and mycophenolate mofetil (1 g/d) for 2 consecutive weeks.

**Figure 6.**
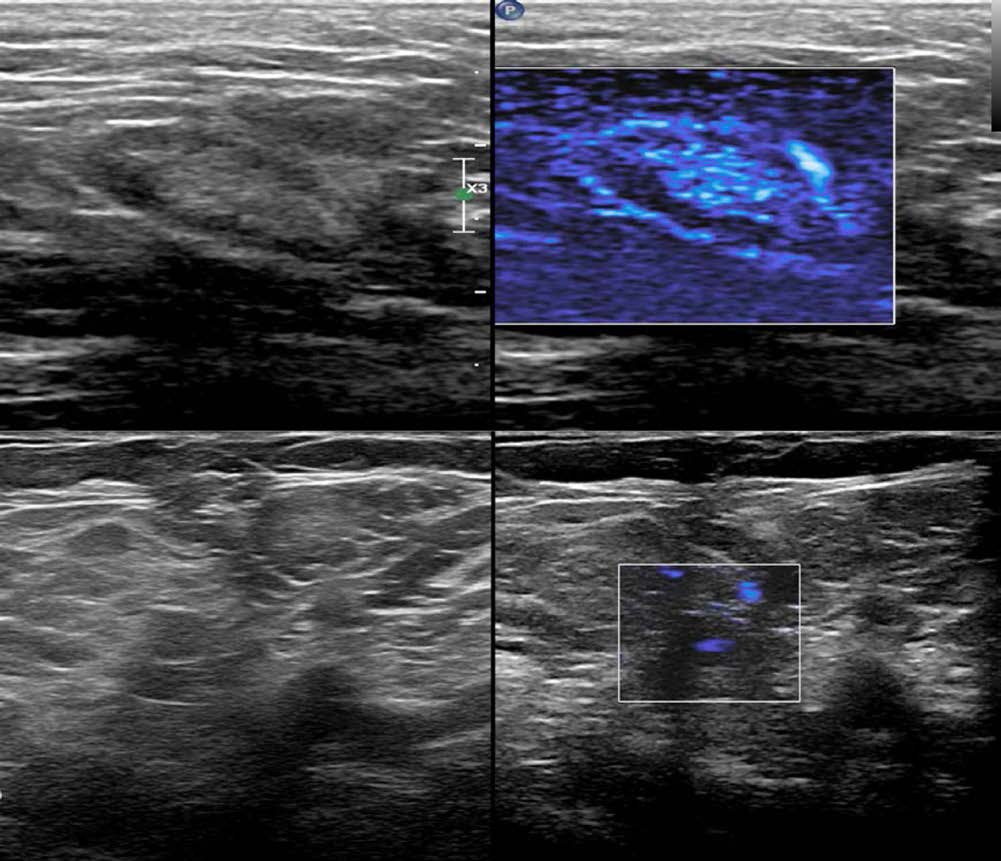
Ultrasonography, showing axillary and inguinal lymph nodes were oval and had normal hilums.

**Figure 7.**
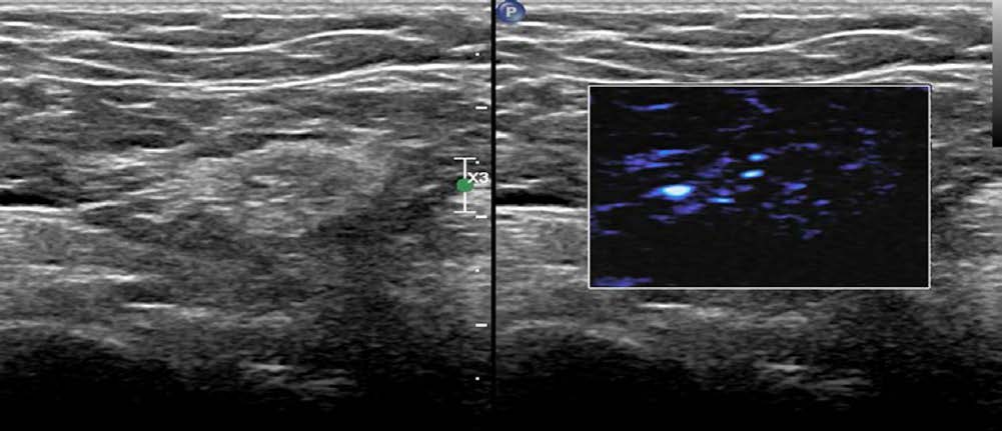
Ultrasonography, showing the axillary lymph nodes had normal hilums.

A subsequent laboratory examination showed that in addition to urine protein all indicators had normalized, and an ultrasound examination showed that axillary lymph nodes shrank (maximum of 1.7 × 0.7 cm), and there was a normal hilum structure. There were similar changes in the inguinal lymph nodes (Fig. 6). Three months later, laboratory examination showed that the urine protein was still high, but color Doppler ultrasonography showed slight enlargement of unilateral axillary lymph nodes, with a maximum size of 1.5 × 0.7 cm, and normal hilum structure (Fig. 7).

## 3. Discussion

The AAV are a group of autoimmune diseases that involve multiple organ systems. A patient may present with constitutional symptoms suggestive of a chronic inflammatory disease (fatigue, weight loss, fever, night sweats, myalgia, or polyarthralgia) or with specific features of end-organ damage.^[6]^ Because almost any part of the body can be affected, the initial symptoms are variable. Lymph node involvement is a rare clinical manifestation of AAV, although some patients have mediastinal and hilar lymph node enlargement based on computed tomography. We presented a rare case of AAV whose initial manifestation was axillary lymphadenopathy.

The axillary lymph nodes mainly drain the breast, upper extremities, and thoracic wall, so diseases in these 3 regions are the main causes of axillary lymphadenopathy. The causes include malignancies, infections, autoimmune disorders, miscellaneous and unusual conditions, and iatrogenic causes. Most axillary lymphadenopathy is nonspecific or reactive in etiology.^[7]^ Reactive lymphadenopathy, the most common cause of lymph node enlargement, is a non-neoplastic and reversible enlargement of lymphoid tissues that is secondary to antigen stimulus, and usually indicates an underlying disease.^[8]^ Some of the major causes are inflammation, viral infection, vaccination, drug reaction, and autoimmune disorders. Our patient initially presented with only enlargement of the axillary lymph nodes, and the ultrasonographic features were suggestive of malignant lymphadenopathy. However, the pathological results strongly suggested reactive hyperplasia, with exclusion of malignant tumor metastasis and lymphoma, so a “watchful waiting” approach was adopted. One year later, the patient presented with inguinal lymph nodes that had similar ultrasonic manifestations, as well as proteinuria and renal damage. These findings led to our diagnosis of AAV.

There are only rare reports of superficial lymphadenopathy in patients with AAV, especially as the initial symptom. We therefore conclude that AAV should be considered in the differential diagnosis when patients present only with enlarged lymph nodes and no other symptoms or signs.^[7]^ Some patients with AAV are negative for ANCA, in which case diagnosis may be based on progression or stage of the disease. However, some patients with ANCA-negative AAV may have ANCAs that cannot be detected by current methods, or ANCAs with as yet undiscovered specificity.^[9]^ The level of ANCAs is a specific and sensitive marker for disease activity and recurrence, although this level does not always parallel the clinical course.^[10]^ Nonetheless, a positive ANCA test greatly enables the early diagnosis of AAV. In our patient, standard corticosteroid and immunosuppressant therapy led to a negative result for anti-myeloperoxidase ANCA and shrinkage of the axillary lymph nodes to normal. Serial change in lymph node size is useful for monitoring response to therapy.^[11]^ We also performed follow-up ultrasonography of our patient’s lymph nodes.

For AAV patients in clinical remission, an elevated ANCA titer should be considered suggestive of disease recurrence, in which case the vigilance and follow-up frequency should be increased. Although corticosteroid and immunosuppressant therapy can alleviate many symptoms, patients with AAV still have a high risk of death. The kidneys are the most commonly affected organ. Renal involvement is initially asymptomatic, but the disease has the potential to progress rapidly and lead to end-stage renal failure in a relatively short time.^[12]^

## 4. Conclusion

Patients with AAV have variable manifestations and early diagnosis can be difficult. Axillary lymphadenopathy as the initial manifestation of AAV is very rare. The details of the case described here may help to improve understanding of the possible initial manifestations of AAV, and help clinicians to make more prompt and accurate diagnosis of AAV so these patients can receive timely treatment that prevents disease progression and improves prognosis.

## Acknowledgements

We thank the patient for providing permission to share her information.

## Author contributions

**Investigation:** Jie Du, Hongyue Wang.

**Validation:** Jie Du, Hongyue Wang.

**Visualization:** Lili Zhang, Hongyu Li, Shuang Li.

**Writing – original draft:** Jie Du, Hongyue Wang, Chao Zhang, Fangfang Sun.

**Writing – review & editing:** Lirong Zhao.
